# Racial/Ethnic Differences in Health Behaviors and Its Roles on Depressive Symptoms among Young Female Adults

**DOI:** 10.3390/ijerph17197202

**Published:** 2020-10-01

**Authors:** Jaewon Lee, Jisuk Seon

**Affiliations:** 1 Department of Social Welfare, Inha University, Incheon 22212, Korea; j343@inha.ac.kr; 2 Department of Social Welfare, Kyungnam University, Changwon 51767, Korea

**Keywords:** health behaviors, food intake, depressive symptoms, female, racial/ethnic disparities

## Abstract

This study explores the role of health behaviors on depressive symptoms across young adult females and differences in the relationship across race/ethnicity. The data come from the National Longitudinal Survey of Youth 1979 Child and Young Adult. Seven hundred and seven non-Hispanic White females, 592 African American females, and 349 Hispanic females were selected. Multiple linear regression and logistic regression analyses were conducted. African American and Hispanic females were more likely to eat fast food than non-Hispanic Whites. African Americans reported that they ate fruit less frequently in comparison with non-Hispanic Whites. Fruit intake was related to lower levels of depressive symptoms. Hispanics moderated the association between fruit intake and depressive symptoms. Females should be encouraged to eat more fruit during young adulthood in order to diminish the likelihood of depressive symptoms. In addition, strategies for promoting healthy behaviors should consider the varied effects of race/ethnicity on depressive symptoms among young female adults.

## 1. Introduction

Affecting more than 300 million people worldwide, mental health problems, especially depressive symptoms, are one of the leading causes of disability. In the U.S., approximately 17 million adults, or almost 7% of the adult population, experienced at least one major depressive episode in 2017 [1]. These episodes are more common during young adulthood [2] and are more prevalent in women [3,4]. Responding to the higher risk of mental health problems among young female adults, and its potential negative consequences, this study focuses on health behaviors that could affect their mental health, such as food intake and preventive healthcare visits, which might interact with each other. Although research has indicated that positive health behaviors, including a greater intake of healthy food (e.g., fruits), visits for preventive healthcare, and help to improve mental health outcomes, little is known about racial/ethnic disparities in the relationships [5,6,7]. This study fills that gap by examining how health behaviors affect mental health among young female adults, and how these relationships differ by race/ethnicity.

Young adulthood is a developmentally important period in the course of life because it is a time when individuals are expected to assume responsibilities for their health behaviors, such as dietary routines and medical check-ups [8]. Some young adults are successful in meeting these challenges and can benefit from practicing healthy behaviors, but others who fail to navigate these life transitions might experience adverse heath trajectories in the future [8,9]. The onset of mental health problems, such as depressive symptoms, typically occurs in young adulthood between the 20 s and 30 s [2]. A recent study based on the National Survey on Drug Use and Health, a nationally representative survey of U.S. adolescents and adults, reported that rates of depressive episodes have drastically increased from 2005 (8.1%) to 2017 (13.2%) among young adults 18–25 [10], indicating that young adults are at a higher risk for depressive symptoms. Such mental health problems are more prevalent in female adults [3]. For example, a U.S. study on the prevalence of mental distress, especially depressive symptoms, reported that more females suffered from depressive symptoms than males in all 50 states [4]. Together, the data suggest that young female adults may be at higher risk for negative mental health outcomes.

Healthy behaviors, including a higher intake of fruits and vegetables, a lower intake of fast food, and regular visits for preventive care are found to be associated with better mental health outcomes [11,12]. For example, a meta-analysis of 21 studies on dietary patterns and depression risk found that healthy dietary patterns, characterized by a high intake of fruits and vegetables, were associated with a decreased risk of depressive symptoms, while unhealthy dietary patterns, characterized by the high consumption of high-fat foods and a low intake of fruits and vegetables, were associated with an increased risk of depressive symptoms [11]. These patterns, showing a positive relationship between healthy eating and better mental health outcomes, were confirmed in a similar way in two systematic reviews on dietary patterns and depressive symptoms. They indicated that a greater intake of fish and vegetables was associated with a lower risk of depressive symptoms [12,13].

As suggested in previous studies, there is strong evidence supporting that adherence to a healthy diet, such as a higher intake of fruits and lower intake of fat, will improve mental health. However, the majority of studies on this topic have focused on middle-aged or older adults [14,15,16]; only a handful of studies have examined the impact of health behaviors on mental health among young female adults, despite their being at higher risk for mental health issues, such as depressive symptoms, compared to older females [17]. For example, Allgöwer and colleagues (2001) surveyed 2091 male and 3438 female university students with an average age of 21.6 years about how their health behaviors—including fruit consumption, eating breakfast, physical exercise, sleep time, and not using sunscreen—affected depressive symptoms in 16 European countries. They found that the lack of eating fruits and breakfast and not using sunscreen were significantly associated with depressive symptoms among female students [18]. Beydoun and Wang (2010) investigated pathways linking socioeconomic characteristics, obesity, depressive symptoms, and lifestyle factors (e.g., dietary intake or physical activity) among 2217 young adults between 20 and 39 years of age using the National Health and Nutrition Examination Survey. They found that major depression disorders were inversely associated with physical activity, but not with dietary intakes among young female adults [19]. This result is contradictory to general findings on the positive relationship between a healthy diet and mental health outcomes. However, another study reported findings comparable to Beydoun and Wang (2010); although fruit and vegetable consumption increased flourishing behaviors (e.g., curiosity, creativity, motivation) among 171 young adults, a randomized controlled trial found no change in depressive symptoms [20]. These inconsistent findings suggest a need for more studies to elucidate the role of a healthy diet in mental health, particularly for young female adults.

Another shortcoming in the previous research on the relationship between health behaviors and mental health is that they often exclude other possible health behavior factors [12], such as the utilization of preventive care, which may also have an effect on mental health outcomes. Knowing that health behaviors are comprised of the interplay among a variety of health-promoting actions, it is necessary to examine other health behaviors such as visits for preventive care (e.g., check-ups, eye exams) and timely, managed treatments that could potentially influence mental health. Within the small number of available studies on health behaviors and mental health among young female adults, very few investigated comprehensive measures of health behaviors (i.e., dietary routines, patterns of visits for preventive care, and treatment) and their roles on mental health outcomes.

For example, Sánchez Villegas and colleagues (2017) examined the relationship between the intake of fast food and commercial baked goods, and depressive symptoms among 8964 college graduates (mean age of females = 35.1 from the original sample characteristics) [21]. Although they asked whether the study participants used screening tests, such as medical and dental check-ups, as well as assessing their fast food and commercial baked goods consumption in relation to depressive symptoms, their results did not report how the screening tests were associated with the risk of depressive symptoms [22]. Peltzer (2004) examined how social support and health behaviors influenced stress and depressive symptoms using a sample of 624 students in South Africa. Among those in the sample, the young females ranged in age from 17 to 39 years (mean age of females = 23.8) and responded about whether they had utilized medical tests in the previous two years, including a physical exam, dentist visit, eye check-up, breast self-exam, or mammogram, and answered some questions about healthy eating and the use of alcohol and substances. While health behaviors, including dental visits, eye check-ups, breast self-exam, mammogram, and healthy eating were negatively associated with depressive symptoms, a physical exam was positively associated with depressive symptoms [23].

Although research on the prevalence of mental health issues, especially depressive symptoms, among young female adults has reported variations across race and ethnicity, the findings were not conclusive. Some studies found that Hispanics and African Americans were more likely to have higher levels of depressive symptoms than Whites [24,25,26]; others, however, found that Whites tended to have higher levels of depressive symptoms than Hispanics or African Americans [27,28]. These disparities may be due to the use of different measures of depressive symptoms and different levels of accessibility to mental health services within the sample [29]. In addition to these reasons, a few other studies have suggested that modifiable health behaviors may also impact racial/ethnicity disparities in the rates of depressive symptoms among young female adults [5,6,7]. For example, first, Balsam and colleagues examined racial/ethnic differences among sexual minority young females (mean age = 20.88) in relation to health behaviors (e.g., alcohol and substance use) and mental health outcomes. They found that levels of depression were the highest in Latin Americans, followed by White Americans, African Americans, and Asian Americans, and there was a significant difference in lower peak drinking among African Americans, compared to White Americans [5]. Second, Mokrue and Acri (2015) investigated how health behavior factors (e.g., use of alcohol and smoking) affected symptoms of depression and anxiety among 567 undergraduate minority students (mean age = 19.89, 73% of the sample were female). They found no racial/ethnic differences in the symptoms of depression across African Americans, Hispanic Americans, Asian Americans, and others. However, the study did not examine racial/ethnic differences in health behaviors [6]. Third, Norris and colleagues (2019) examined sexual risk and health behaviors (e.g., alcohol and substance use) and depressive symptoms among 466 young females aged 18–29 years. They found that Hispanic females reported significantly higher levels of depressive symptoms than non-Hispanic Whites, and Hispanic and African American females were less likely to use alcohol and cigarettes but more likely to use water pipe tobacco than their White counterparts [7].

Even though these studies contributed to building knowledge on racial/ethnic disparities in health behaviors and mental health among young female adults, they focused on certain types of health behaviors, such as substance use, not considering the potential role of other health behaviors on mental health, such as preventive healthcare visits. In addition, they did not examine how such health behaviors predicted mental health outcomes [5,6,7]. In addition, generalizability is limited because they examined a unique sample who belong to a sexual minority [5] or excluded a major racial/ethnic group (e.g., Whites) in the analysis model [6].

Based on identified gaps in the previous research, this study will examine racial/ethnic differences in health behaviors (e.g., food intake, preventive healthcare visits) among young female adults using a nationally representative sample (Research Question 1). This study will also explore how health behaviors influence mental health outcomes and how this association differs by race/ethnicity among young female adults (Research Questions 2 and 3).

## 2. Materials and Methods

### 2.1. Target Sample

The data come from the National Longitudinal Survey of Youth 1979 Child and Young Adult (NLSY79-CHYA), which was administrated by the United States Department of Labor. In other words, the sample in this study is representative of Americans. This study uses the latest wave of the NLSY79 CHYA, from 2012, which includes interviews with 11,512 children. The dataset provides information on health behaviors, such as food intake and preventive healthcare visits, as well as mental health problems such as depressive symptoms. Participants who were not interviewed or refused to be interviewed regarding depressive symptoms were excluded from this study. Since this study focuses on females and young adults, the final sample consists of only females aged 21 years and older. Individuals who were male and younger than 21 years old were excluded in this study. 707 non-Hispanic White females, 592 African American females, and 349 Hispanic females were selected; the mean age of the sample was 27.4 years. Since the NLSY79-CHYA is a public access database, an approval from the Institutional Review Board is not necessary for the proposed study, and this research is considered non-human subject study. In addition, there is no identifiable information in the dataset.

### 2.2. Measures

Mental health. In this study, mental health indicates levels of depressive symptoms. The Center for Epidemiologic Studies Depression Scale (CES-D) was used to measure levels of depressive symptoms. This measurement consists of eleven items, rated on a four-point Likert-type scale in which response options range from 0 “rarely or none of the time (<1 day)” to 3 “most or all of the time (5–7 days).” Respondents were asked, “I felt I could not shake off the blues,” “I felt depressed,” “I felt lonely,” “I felt sad,” “I felt life was not worth living,” “I was happy,” and the like. The response “I was happy” was reverse-coded, and total scores were computed as the mean of all items, with higher scores indicating higher levels of depressive symptoms (Mean = 0.50; SD = 0.47). The CES-D is highly correlated with other measurements for depressive symptoms [30].

### 2.3. Health Behavior

#### 2.3.1. Food Intake

Fruit. The respondents were asked about the frequency of fruit intake. They rated how often they eat fruit with the following options: “do not eat any fruits, one to three times per week, four to six times per week, one time per day, two times per day, three times per day, four or more times per day.” The answer options were classified into two categories: those who never eat any fruit (code = 0) and those who eat fruit at least one time a week (code = 1).

Fast Food. Respondents reported the frequency of fast food intake. They answered the following statement: “How many times have you eaten fast food in the past week?” Higher scores mean more frequent fast food intake.

#### 2.3.2. Preventive Healthcare Visits

Eye exam. Respondents were queried about their eye exams. They were asked when they visited doctors for their routine eye exam, with seven response options: “Less than one month ago, one to three months ago, four to six months ago, seven to eleven months ago, less than two years ago, two or more years ago, and never saw a doctor for their eye exam.” The respondents were classified into two groups: those who had never visited a doctor for an eye exam (code = 0) and those who had met with a doctor for an eye exam more than once (code = 1).

Check-up. The respondents answered the following question, “When did you see a doctor for your routine health check-up?” with seven response options: “Less than one month ago, one to three months ago, four to six months ago, seven to eleven months ago, less than two years ago, two or more years ago, and never saw a doctor for their check-up.” They were coded with “1” if they had experienced check-ups more than once, while those who never visited a doctor for a health check-up were coded as “0”.

Control variables. Demographics and socioeconomic status were included as control variables in this research as follows: age, race/ethnicity, family size, marital status, education, income, employment, and residence. These variables were related to depressive symptoms, and we controlled these factors in this study. Age was measured in years and treated as a continuous variable. Race/ethnicity had three categories: non-Hispanic Whites, African Americans, and Hispanics. Family size, the number of family members, was used as a continuous variable. Marital status was a binary variable: Yes or No. Those who were currently married were regarded as married, while those who were not married were considered non-married couple. Education was a binary variable: individuals having less than a bachelor’s degree (code = 0) (e.g., high school, middle school, etc.) and more than a bachelor’s degree (code = 1) (e.g., bachelor’s, master’s, PhD, MD, etc.). Employment was a binary variable: employed (code = 0) and unemployed (i.e., unemployed, out of labor force, or in active forces, code = 1). Residence was a binary variable: urban (code = 0) and rural (code = 1). In addition, the study included treatment for illness, which may influence mental health. This variable was classified into two categories: individuals who had never seen a doctor for the treatment of illness (code = 0) and those who visited a doctor for the treatment of illness more than once (code = 1).

### 2.4. Statistical Analysis

Racial/ethnic disparities in health behaviors were tested using multiple linear regression and logistic regression analyses. Multiple linear regression was also conducted to test the association between educational attainment and mental health as well as the moderating roles of race/ethnicity. We entered demographics and socioeconomic status first and then progressively adjusted for health behaviors and the race/ethnicity × food intake interaction terms.

## 3. Results

### Descriptive Statistics

As shown in Table 1, the average age of the female participants was about 27 years old, and roughly one-fifth of them reported that they were married. About one-fourth had received a higher education (more than a bachelor’s degree), and about 68% of the participants were employed. Their average income was about 17,000 USD. They ate fast food about 1.5 times per week and about 93% of them ate fruit at least one time a week. More than 95% of the female participants had both undergone check-ups more than once and visited a doctor for the treatment of an illness more than once in the past year. However, only about 88% of the females had met with a doctor for an eye exam more than once in the previous year. The average depressive symptoms score across females was 0.5.

Findings for Research Question 1: Are there racial/ethnic disparities in health behaviors (food intake and preventive healthcare visits) among young adult females?

There were racial/ethnic disparities in food intake. Both African American and Hispanic females were more likely to eat fast food than non-Hispanic Whites (β = 0.51, *p* < 0.001; β = 0.50, *p* < 0.001) (Table 2). African Americans reported that they ate fruit less frequently in comparison with non-Hispanic Whites (β = −0.83, *p* < 0.01) (Table 2). On the other hand, there were no racial/ethnic disparities in preventive healthcare visits, such as check-ups and eye exams (Table 3).

Findings for Research Questions 2 and 3: How do health behaviors influence mental health among young adult females and does the relationship differ across race/ethnicity?

**Figure 1:**
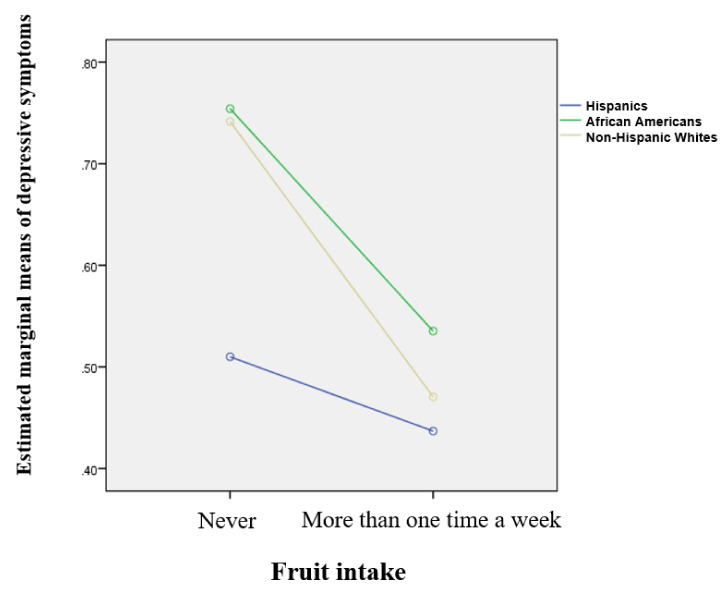
Roles of fruit intake and race/ethnicity on depressive symptoms.

Table 4 showed that there was an association between health behaviors and mental health across young adult females. Model 1 reveals that Hispanic females were less likely to suffer from depressive symptoms compared to non-Hispanic White females (β = −0.08, *p* < 0.05). Older age was significantly associated with greater risks of depressive symptoms (β = 0.01, *p* < 0.001). Young adult females who were married, had achieved educational attainment, were employed, and earned more income were less likely to be depressed (β = −0.17, *p* < 0.001; β = −0.07, *p* < 0.05; β = −0.07, *p* < 0.05; β = −1.49, *p* < 0.05). As health behaviors were entered into model 2, Hispanic females still tended to be at a lower risk for depressive symptoms compared to non-Hispanic White females (β = −0.08, *p* < 0.05) and other demographics and socioeconomic status (SES) factors remained significant. Among health behaviors, fruit intake was significantly related to lower levels of depressive symptoms (β = −0.19, *p* < 0.001). As shown in model 3, interaction effects were found, indicating that Hispanics moderated the association between fruit intake and depressive symptoms (β = 0.28, *p* < 0.05). Figure 1 indicates the differences across racial/ethnic groups. All racial/ethnic groups demonstrated that levels of depressive symptoms decreased if they were to eat fruit more than one time a week compared to those who never did. However, the roles of fruit intake on depressive symptoms were slight in Hispanic females (0.51 vs. 0.44), while levels of depressive symptoms across non-Hispanic White females were greatly influenced by fruit intake (0.74 vs. 0.47).

## 4. Discussion

The primary purpose of this study was to explore the roles of health behaviors on mental health among young female adults in the United States and identify racial/ethnic disparities in the relationships. Given that females are likely to be at greater risk for mental health problems compared to men, and that health behaviors influence mental health, this study focused on young female adults over 21 years of age and the roles of two different dimensions of health behaviors (e.g., food intake and preventive healthcare visits) on mental health. This study contributes to understanding racial/ethnic disparities in mental health among young female adults. Findings in this study indicated racial/ethnic disparities in health behaviors and mental health. Furthermore, race/ethnicity moderated the relationship between food intake and mental health across young female adults: the association between fruit-intake and depressive symptoms was stronger for non-Hispanic White females than for Hispanic females; depression had less influence on fruit intake in Hispanic females, while depression among non-Hispanic White females had a lot of influence on fruit intake.

This study demonstrated that there were racial/ethnic disparities in health behaviors, which is consistent with previous studies [5,7]. Compared to previous findings, this study included two different dimensions of factors explaining health behaviors (e.g., food intake, preventive healthcare visits). Both African American and Hispanic young female adults tended to eat more fast food and less fruit compared to non-Hispanic White females. Economic resources might be the first parameter to consider for encouraging a habit of healthy food intake. Those with fewer economic resources may have trouble accessing healthy food, such as fruit, and find it easier to eat junk food, such as fast food [31]. Financial hardship may, therefore, lead to poor health behaviors for food intake. There were racial/ethnic differences in economic activities and resources, indicating that minorities were more likely to have fewer economic resources when compared with non-Hispanic Whites [32]. In this study, both African American and Hispanic females were less likely to visit a doctor for the treatment of an illness. This may also be related to their economic resources and to health insurance. To receive treatment, they would have to pay for a doctor’s services; those living in poverty or with low incomes are often not able to pay for healthcare, especially if they do not have health insurance. In general, as those with fewer economic resources (e.g., the unemployed) and minorities are more likely to occupy a lower economic status [32], it is evident that African Americans and Hispanics tend to not see a doctor for treatment if they are sick. Since this study focused on young female adults, findings in this study contribute to understanding racial/ethnic disparities in health behaviors across females in young adulthood, demonstrating that minority females are more exposed to poor health behaviors compared to their counterparts. Given that there are positive roles of better health behaviors to individuals and society [33] and minority females have poor health behaviors in both food intake and preventive healthcare visits, encouraging them to develop healthy habits and providing more opportunities for economic resources to increase accessibility to healthcare visits should be considered.

Affirming the findings from other research [11,12,13], this study demonstrated that health behaviors are related to depressive symptoms. Findings in this study provide further empirical evidence for understanding that relationship by focusing on young adult females. Among diverse factors about health behaviors in this study, fruit intake affected levels of depressive symptoms while fast food did not. Generally, junk food, including fast food, is regarded as food that influences poor health [34]. However, the impact may be limited to physical health rather than mental health [35]. Many people have enjoyed eating fast food due to its addictive taste and affordable price, and it is common for people to eat fast food regardless of socioeconomic status, often making individuals feel happy in the moment. As such, eating fast food may not be related to mental health. However, fruit intake is not aligned with eating fast food, because those living in low income or poverty conditions tend to not buy fruit because of the financial burden [36,37]. They may rather prefer to purchase other foods or goods for their daily lives. In other words, the ability to buy fruit may be associated with levels of depressive symptoms. Likewise, findings in this study support that individuals who were employed and earned more income were less likely to suffer from depressive symptoms. Another mechanism, such as economic ability, should be considered to understand roles of fruit on depressive symptoms. Since fruit itself has the role of improving poor mental health [11] and females are more likely to be at greater risk for mental health problems compared to males [3], it is imperative that females should be encouraged to eat more fruit during young adulthood in order to diminish the likelihood of depressive symptoms. On the other hand, young female adults with depressive symptoms may not often eat fruits because of their lethargy. Thus, it is also important for depressed young female adults to be encouraged to consume more fruits as fruit intake has an effect on their health. While other studies have not included a comprehensive concept of health behaviors [38,39,40], this study classified health behaviors in two dimensions to address both aspects of health behaviors: food intake and preventive healthcare visits. In this study, preventive healthcare visits did not influence depressive symptoms. Eye exams and check-ups may not influence current mental health status, because the purpose of the behavior is to prevent poor physical health in the future. This study confirms that food intake is more important to depressive symptoms than preventive healthcare visits. It adds to our understanding of what types of health behaviors are more important to depressive symptoms across young adult females.

The study found that depressive symptoms in Hispanic young female adults was less influenced by fruit intake compared to non-Hispanic Whites for whom fruit intake had a great influence on depressive symptoms levels. This finding has advanced our understanding within the Hispanic paradox, particularly relating to mental health and the roles of food intake on depressive symptoms. According to the Hispanic paradox, Hispanics in the United States are less likely to have poor health and higher rates of mortality compared to other populations due to their familism and ethnic identity [41]. Hispanics have also shown fewer mental health problems in comparison with their counterparts [27,28]. So, even though females generally have a greater risk of depressive symptoms compared to men, this study demonstrated that Hispanic females still had lower levels of depressive symptoms compared to non-Hispanic White females. This may explain why the roles of fruit intake did not significantly influence levels of depressive symptoms across Hispanic females. By the same token, it is generally believed that underprivileged groups may have a greater likelihood of suffering from poor health [42,43], but this study displayed the Hispanic paradox in depressive symptoms. Because of the paradox, young adult non-Hispanic White females should be more protected from depressive symptoms compared to young adult Hispanic females.

### Limitations

This study demonstrated the comprehensive concept of health behaviors by including two different perspectives: food intake and preventive healthcare visits. However, other dimensions should be considered, such as regular exercise. This is a limitation that arises from using nationally representative surveys, because they only offer a limited number of factors. A more extensive array of factors regarding health behaviors would have helped further the understanding of health behaviors and of how they influence depressive symptoms. Since this study focused on young adult females, most of the participants were aged between 21 and 30 years old. However, given that females being at greater risk of suffering from mental health problems than males is a continuous issue over time [2] and health behaviors are closely related to mental health [11], it would be beneficial to explore whether the same findings emerge when health behaviors and depressive symptoms are measured in adolescence and middle/older adulthood. In addition, future studies need to consider other racial/ethnic groups, such as Asians, to show more comprehensive effects of racial/ethnic disparities in the relationship between health behaviors and mental health. Further, one of the possible indicators influencing depressive symptoms is health insurance, but this study could not include the variable due to the limitation of using secondary data. This study does not address the gender effect because the primary focus is to investigate racial and ethnic disparities in the relationships between healthy behaviors and depressive symptoms among only young female adults. Gender differences may be an interesting topic in the relationships across young adults. Thus, we suggest that future studies include both young male and female adults to see if there is a gender difference. Lastly, this study was conducted based on a cross-sectional approach. It makes it impossible to interpret a cause and effect, so we recommend that future studies consider a longitudinal analysis to show causal relations between healthy behaviors and depressive symptoms among young female adults.

## 5. Conclusions

Minority females should be encouraged to develop healthy habits and have more chances to access healthcare visits. Among types of health behaviors, poor food intake rather than preventive healthcare visits is associated with depressive symptoms in young women. According to the Hispanic paradox, this association is stronger for non-Hispanic White women than for Hispanic women. Thus, it is important to increase fruit intake in non-Hispanic White women in order to address their depressive symptoms.

## Figures and Tables

**Table 1:** Descriptive statistics for variables included in the study.

Variable	Total
	(*n* = 1648)
	% or Mean (SD)
Depressive symptoms	0.50 (0.47)
Healthy behaviors	
Food intake	
Fast food	1.52 (1.93)
Fruit	92.8%
Preventive healthcare visit	
Eye exam	87.6%
Check-up	98.7%
Demographics	
Age	27.40 (5.09)
Race (non-Hispanic White)	42.9%
Family size	3.51 (1.60)
Marriage	21.7%
Higher education	26.5%
Income	1.70 (2.16)
Employment	67.9%
Urban residence	84.3%
Treatment	96.3%

Notes. The real values of net worth and income should be multiplied by 10,000.

**Table 2:** Regression results of unstandardized coefficients (standard error) predicting fast food and logistic regression coefficients (odds ratio) predicting fruit intake.

Variables	Food Intake							
	Fast Food				Fruit			
	Model 1		Model 2		Model 1		Model 2	
(Constant)	1.16 (0.06)		0.89 (0.27)		3.11 (0.20)		3.43 (0.72)	
Race/Ethnicity								
African American	0.56 (0.09)	***	0.51 (0.10)	***	−1.00 (0.37)	***	−0.83 (0.44)	**
Hispanic	0.51 (0.11)	***	0.50 (0.11)	***	−0.20 (0.82)		−0.09 (0.92)	
Demographics								
Age			0.10 (0.10)				−0.02 (0.98)	
Family size			0.02 (0.03)				0.03 (1.03)	
Marriage			−0.08 (0.10)				0.27 (1.31)	
Higher education			−0.38 (0.10)	***			0.83 (2.29)	*
Income			1.97 (0.00)				0.00 (1.00)	
Employment			0.23 (0.09)	*			−0.15 (0.86)	
Urban residence			−0.18 (0.11)	+			−0.29 (0.75)	

Note. + *p* < 0.10. * *p* < 0.05. ** *p* < 0.01. *** *p* < 0.001.

**Table 3:** Logistic regression coefficients (odds ratio) of predicting preventive healthcare visit.

Variables	Preventive Healthcare Visit			
	Check-Up		Eye Exam	
	Model 1	Model 2	Model 1	Model 2
(Constant)	4.20 (0.37)	1.15 (1.72)	2.05 (0.13)	2.23 (0.55)
Race/Ethnicity				
African American	0.23 (1.25)	0.06 (1.07)	−0.20 (0.82)	−0.14 (0.87)
Hispanic	0.77 (2.15)	0.60 (1.82)	−0.07 (0.93)	0.02 (1.02)
Demographics				
Age		0.07 (1.07)		−0.01 (0.99)
Family size		0.13 (1.14)		−0.06 (0.94)
Marriage		0.37 (1.45)		0.12 (1.13)
Higher education		0.32 (1.37)		0.02 (1.02)
Income		0.00 (1.00)		0.00 (1.00)
Employment		0.26 (1.29)		0.20 (1.22)
Urban residence		0.45 (1.57)		−0.12 (0.89)

**Table 4:** Regression results of unstandardized coefficients (standard error) predicting depressive symptoms.

Variables	Depressive Symptoms					
	Model 1		Model 2		Model 3	
(Constant)	0.30 (0.08)		0.52 (0.15)		0.57 (0.15)	
Race/Ethnicity						
African American	0.03 (0.03)		0.02 (0.03)		0.01 (0.03)	
Hispanic	−0.08 (0.03)	*	−0.08 (0.03)	*	−0.36 (0.13)	**
Demographics						
Age	0.01 (0.00)	***	0.01 (0.00)	***	0.01 (0.00)	***
Family size	−0.00 (0.01)		−0.00 (0.01)		−0.00 (0.01)	
Marriage	−0.17 (0.03)	***	−0.17 (0.03)	***	−0.17 (0.03)	***
Higher education	−0.07 (0.03)	*	−0.06 (0.03)	*	−0.06 (0.03)	+
Income	−1.39 (0.00)	*	−1.45 (0.00)	*	−1.44 (0.00)	*
Employment	−0.07 (0.03)	**	−0.08 (0.03)	**	−0.08 (0.03)	**
Urban residence	−0.01 (0.03)		−0.00 (0.03)		−0.00 (0.03)	
Treatment	0.00 (0.07)		0.01 (0.07)		0.01 (0.07)	
Health behaviors						
Food intake						
Fast Food			0.02 (0.01)	+	0.01 (0.01)	
Fruit			−0.19 (0.05)	***	−0.23 (0.05)	***
Preventive healthcare visit						
Check-up			−0.05 (0.11)		−0.06 (0.11)	
Eye exam			−0.01 (0.04)		−0.01 (0.04)	
Fast food × Hispanics					0.00 (0.02)	
Fruit × Hispanics					0.28 (0.13)	*

Note. + *p* < 0.10. * *p* < 0.05. ** *p* < 0.01. *** *p* < 0.001.
